# Mediterranean Species of *Caulerpa* Are Polyploid with Smaller Genomes in the Invasive Ones

**DOI:** 10.1371/journal.pone.0047728

**Published:** 2012-10-22

**Authors:** Elena Varela-Álvarez, Amelia Gómez Garreta, Jordi Rull Lluch, Noemi Salvador Soler, Ester A. Serrao, María Antonia Ribera Siguán

**Affiliations:** 1 CCMAR, CIMAR – Laboratório Associado, Universidade do Algarve, Gambelas, Faro, Portugal; 2 Laboratori de Botànica, Facultat de Farmàcia, Universitat de Barcelona, Barcelona, Spain; 3 Facultad de Educación, Universidad Autónoma de Chile, Temuco, Chile; University of Oxford, United Kingdom

## Abstract

*Caulerpa* species are marine green algae, which often act as invasive species with rapid clonal proliferation when growing outside their native biogeographical borders. Despite many publications on the genetics and ecology of *Caulerpa* species, their life history and ploidy levels are still to be resolved and are the subject of large controversy. While some authors claimed that the thallus found in nature has a haplodiplobiontic life cycle with heteromorphic alternation of generations, other authors claimed a diploid or haploid life cycle with only one generation involved. DAPI-staining with image analysis and microspectrophotometry were used to estimate relative nuclear DNA contents in three species of *Caulerpa* from the Mediterranean, at individual, population and species levels. Results show that ploidy levels and genome size vary in these three *Caulerpa* species, with a reduction in genome size for the invasive ones. *Caulerpa* species in the Mediterranean are polyploids in different life history phases; all sampled *C. taxifolia* and *C. racemosa* var. *cylindracea* were in haplophasic phase, but in *C. prolifera*, the native species, individuals were found in both diplophasic and haplophasic phases. Different levels of endopolyploidy were found in both *C. prolifera* and *C. racemosa* var. *cylindracea*. Life history is elucidated for the Mediterranean *C. prolifera* and it is hypothesized that haplophasic dominance in *C. racemosa* var. *cylindracea* and *C. taxifolia* is a beneficial trait for their invasive strategies.

## Introduction

Green algae of the genus *Caulerpa* J. V. Lamouroux (Chlorophyta, Bryopsidophyceae, Caulerpaceae) have the capacity to propagate clonally by fragmentation and often show invasive behavior when introduced beyond their native ranges, particularly as competitors of seagrasses [1], 2,3. In the last two decades, the genus *Caulerpa* has been attracting considerable research attention in the Mediterranean Sea, where two tropical *Caulerpa* species, *Caulerpa taxifolia* (M. Vahl) C. Agardh and *Caulerpa racemosa* (Forsskål) J. Agardh, have spread into areas formerly occupied by seagrasses, also co-occurring with indigenous *Caulerpa prolifera* (Forsskål) J.V. Lamouroux, which is distributed worldwide. In 1984, *C. taxifolia* was accidentally released into coastal waters of the Mediterranean Sea in Monaco, and spread along the coasts of France, Italy, Spanish Balearic Islands, Croatia, Egypt and Tunisia, reaching nearly 131 km^2^ of subtidal area [1], [4]. This species had also reached Californian coasts in the USA [5]. The potential impact of *C. taxifolia* invasions on biodiversity includes loss of seagrass beds, effects on local fisheries, and general negative effects on the coastal ecosystem [6], all of which have been heavily popularized by the media [7]. The sources of introduction and propagation of *C. racemosa* in the Mediterranean appear more complex, partly because this species includes several distinct strains, which may be distinct species [8], [9]. *C. racemosa* has been considered an introduction in the Mediterranean from the Red Sea via the Suez Channel, but a different variety of *C. racemosa* (var. *cylindracea* (Sonder) Verlaque, Huisman and Boudouresque) has been reported as introduced from Australia in the early 1900 s [10] and is now also detected in the Atlantic, where it has been spreading in the Canary Islands since the late 1990 s [11].

Caulerpales present a coenocytic anatomy: they have no internal cell membranes separating the nuclei within the continuous cytoplasm, and have numerous internal trabeculae (branching ingrowths of the wall). Individuals of *C. taxifolia* have been found to reach 2.8 m, the largest known single cells [12]. Only a few green algae and fungi have this unusual structure. Despite growing concern about the problems that may be caused by proliferation of exotic *Caulerpa* species, little is known about their reproductive biology. The maintenance and spread of *Caulerpa* populations may take place by clonal and/or sexual reproduction, a poorly understood question (but see [13]). Sexual reproduction does occur in *C. taxifolia* as a stochastic event, although rare and apparently absent in the invasive Mediterranean strain, as shown using nuclear and cytoplasmic sequences [14]. At the global biogeographical scale, *C. taxifolia* is a complex of genetically and ecologically differentiated sibling species [14], [15]. It has been suggested that *C. taxifolia* might spread mainly clonally in the Mediterranean, in contrast with *C. racemosa,* where sexual recombination results in hybrid strains among its varieties [16]. Whether any such life history traits are related to invasiveness in these two species has not been elucidated, neither have these questions been addressed for the Mediterranean native *C. prolifera.* There are other cases of invasive populations of *Caulerpa,* such *as* seen recently in parts of South East Florida [17], [18] and in the Azores [19]. It seems that the genus *Caulerpa* is a typical case where, if the species reach localities outside their typical geographical range, they generally become invasive. The roles of sexual and asexual reproduction and of possible diploid versus haploid generations in the capacity for colonization and persistence of *Caulerpa* species are unknown, yet this is basic to understand the invasiveness of species and strains.

### Unclear Life Histories in Caulerpales

A major problem in understanding the traits that lead to frequent invasiveness in species or strains of the genus *Caulerpa* is the conflicting evidence concerning basic traits of their life cycle and the large controversy on the ploidy level of each phase in the life cycle. While some authors claimed that the plant found in nature corresponds to the macroscopic phase of a haplodiplobiontic life cycle with a heteromorphic alternation of generations [20], [21], other authors claimed a diploid or haploid life cycle with only one diploid or haploid generation involved [8], [13], [22], [23], [24], [25], [26], [27], [28], [29]. Thus, despite their ecological and economical importance, the life cycle of the *Caulerpa* species is still to be resolved. Besides, the genus has been reported as dioecious by some authors [30], [31], [32], [33], [34], [35], [36] and monoecious by others [20], [23], [25], [26], [37]. Even within the same species, *C. prolifera*, specimens have been recorded as dioecious [32] and monoecious [20]. However, it is interesting to note that despite all of these contradictory studies, they all report a common characteristic: variability in nuclear size.

### Polyploidy and Genome Sizes in Invasive Species

Polyploidy or genome doubling has been a powerful process in plant evolution [38]. Polyploids occur with greater frequency among invasive plants than among angiosperms in general. There is some evidence that invasive behavior and spread of alien species may be positively correlated with ploidy level [39]. This hypothesis has been tested in some species (e.g. *Rubus alceifolius* Poir. (Rosaceae) [40], or the genus *Spartina* Schreb. (Poaceae) [41]. In general, screening different life stages for ploidy has the potential to identify specific stages where ploidy-level differences may impact fitness [42].

The capacity to adapt and evolve in a changing ecosystem can be a consequence of genome sizes (at least partly). Species with larger genomes have slower than average rates of diversification [43], [44]. The influence of genome size on life strategy in invasive species has been reported as one of the key factors for being a successful invasive weed. Influencing traits such as rapid establishment, short minimum generation time and faster and larger production of seeds seems to correlate with low DNA contents [45]. Although both polyploidy and genome size variation are detected in invasive species, the relation between these in invasive life cycles has been relatively unexplored, but could offer insights into mechanisms of invasions.

### Hypothesis and Aims

The aim of this study was to provide insights into the traits that might influence the success of invasive versus native species of *Caulerpa* in the Mediterranean Sea, by elucidating their ploidy level and genome size for each life history phase. The main objective is to assess the hypothesis that invasive *Caulerpa* species will have higher ploidy levels and smaller genome sizes than the native species, as these are trends reported for other invasive species in general. A second objective is to use the evidence from ploidy levels encountered in nature to construct hypothetical models for the life histories of Mediterranean *Caulerpa* species, as a contribution to solve all the controversy and contradictory previous studies related to life histories in the genus *Caulerpa*. These goals were achieved by estimating genome sizes and ploidy levels using measurements of nuclear DNA contents in the three species of *Caulerpa* occurring in the Mediterranean, at individual, population and species level. This case study is an example of how modulating traits related to DNA contents might be associated to successful invaders.

## Materials and Methods

“The locations for plant collections in this study were not privately-owned or protected in any way, so no specific permissions were required for these locations/activities; also none of the species used in this study involve endangered or protected species”.

### Algal Material

**Figure 1:**
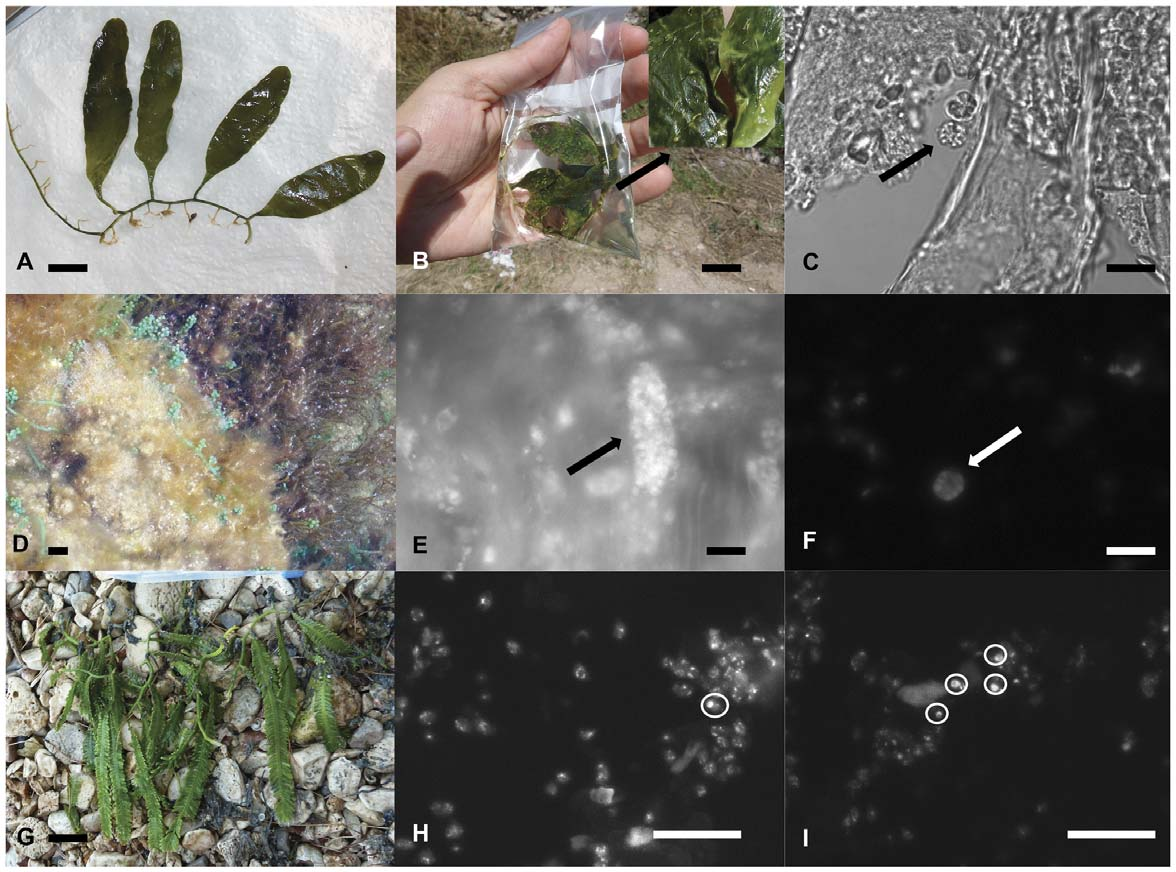
Sterile, fertile and nuclei in *Caulerpas* from the Mediterranean area. Sterile fronds of *C. prolifera*, *C. racemosa* var. *cylindracea* and *C. taxifolia* from the Mediterranean (A, D, G). Gametogenesis in *C. prolifera*: *C. prolifera* with extrusion papillae, mucilage is released from the discharges tubes (B). Optical microscope view and DAPI-stained spherical gametangia containing 8 gametes (C, F). DAPI stained gametangial sacs containing a large number of gametes (E). DAPI-stained nuclei (circular areas point the nuclei), chloroplast circular DNA is also visible (H, J). Scale bars: in A, B, D, G = 1 cm; in C = 10 µm; in E, H, I = 4 µm; in F = 5 µm.

Vegetative fronds of *C. prolifera, C. racemosa* var. *cylindracea* and *C. taxifolia* and also reproductive fronds of *C. prolifera* were collected from the Mediterranean area (Spain and France). This alga consists of three thalli portions: fronds, stolons and rhizoids. The stolons are the tubes from which upright fronds arise, which vary in shape depending of the species studied. The alga is attached to the substrate by thin rhizoids, which lack chloroplasts (Fig. 1). Algal material corresponding to different parts of thalli (fronds, stolons and rhizoids) was preserved in Carnoy fixative and stored in 70% ethanol [35], [36].

A total of 10 algae samples were analyzed: a) for *C. prolifera*, 2 fertile individuals (1 from Cala D’Or, Majorca, Spain, and 1 from Cases d’Alcanar, Catalonia, Spain), and 5 sterile samples (1 from Cases d’Alcanar, Catalonia Spain; 1 from Cala D’Or, Majorca, Spain, 2 from Lo Pagán, Murcia, Spain; 1 from Amerador, Alicante, Spain); b) for *C. racemosa* var. *cylindracea,* 2 individuals from Santa Pola, Alicante, Spain, and c) for *C. taxifolia,* 1 individual from Villefranche-sur-Mer, France. Number of nuclei measured per thallus portion, population, species and location are given in Table 1.

**Table 1:** Genome size variation and ploidy levels.

Ploidy level			2Cx	3Cx	4Cx	6Cx		8Cx	12Cx	16Cx	32Cx
Genome size approximation (µm^2^)			0.5	0.7	1	1.5		2	3	4	8
		Nuclei Number (**)									
R *-C. prolifera*	Gametes(*)	26 (18∶8)	0.49 (0.08)		0.88 (0.08)			–		–	
	R -Frond	870 (517∶293∶51∶9)	0.51 (0.10)		0.97 (0.21)			1.96 (0.37)		3.72 (0.75)	
*C. prolifera*	Frond	452 (389∶38∶22∶3)			1.07 (0.37)			2.29 (0.23)		3.67 (0.3)	7.54 (1.29)
	Stolon	245 (238∶7)			0.86 (0.23)			1.71 (017)		–	–
	Rhizoid	196 (164∶32)			1.00 (0.28)			1.75 (0.31)		–	–
*C. racemosa* (+)	Frond	434 (390∶43∶1)		0.70 (0.18)		1.33 (0.14)			2.94		
	Stolon	409 (380∶29)		0.76 (0.18)		1.47 (0.25)					
	Rhizoid	205 (192∶13)		0.57 (0.16)		1.34 (0.29)			–		
*C. taxifolia*	Frond	152 (123∶29)	0.55 (0.11)		1.12 (0.14)				–		
	Stolon	167 (157∶10)	0.54 (0.15)		1.14 (0.12)						
	Rhizoid	nt	–		–						

Genome size variation and ploidy levels in different thalli portions of the three *Caulerpa* species from the Mediterranean Sea. Number in brackets represented standard deviation. (*) Gametes found inside the papillae. (**) Number of nuclei in each thalli portion analyzed, in brackets number of nuclei that failed into each class. (+) *C. racemosa* var. *cylindracea*. nt (not tested). R –*C. prolifera* (reproductive *C. prolifera*). R-Frond (reproductive frond).

### Flow Cytometry vs. Microfluorometric Analyses

Flow cytometry and microfluorometric analyses have been used to directly compare relative genome sizes and ploidy levels of species over time. Although flow cytometry is a rapid technique (100–500 cells/sec) highly used for microalgae (e.g. [46], [47], [48], [49]) and some macroalgae [50], [51], microspectrophotometry followed by image analyses allows the user to differentiate and curate every single data unit produced (nuclei can be checked by optical microscopy before the fluorescence microscope), thus more rigorous despite having the drawback of being a much slower technique. Most genome sizes (C-values) studied in macroalgal groups were assessed with this last technique [52]. In the case of *Caulerpa* species the presence of intra- and extracellular bacteria [53] and the difficult localization and isolation of nuclei because of their small size and of intricate cell wall thickenings [54], [55], [56] discourage flow cytometry measurements in favor of using microspectophotometric analyses.

### Microfluorometric Analysis, Nuclear DNA Content Estimation and Assignment of Ploidy Level

Samples were rehydrated in water and softened in 5% w/v EDTA for 12–48 h. Specimens of each species (fronds, stolons and rhizoids) were transferred to coverslips treated with subbing solution and then air dried and stained with 0.5 mg/mL 4^I^-6-diamidino-2-phenylindole (DAPI; Sigma Chemical Co., St. Louis, MO 63178). Nuclear DNA content parameters, such as Area (µm^2^), Rfu (Relative fluorescence units), Total Area Average Intensity and Total Intensity, were estimated from microspectrophotometry and image analysis. These estimate followed procedures specified previously [21], [57], (modified after [58]), using a cooled CCD Miramax RTE 782-Y high-performance digital camera on a Leica DMRB fluorescence microscope, and analyzed using MetaMorph software (Molecular Devices, Toronto, ON, Canada). Attempts to quantify the DNA content in picograms were made by comparing total fluorescence intensity values from our samples with those for chicken erythrocytes with a known DNA content of 2.4 pg [59]. However the intensity of the *Caulerpa* nuclei was much inferior to the chicken nuclei, so photos could not be taken at the same exposure/intensity, and consequently determination of pg was not possible. Instead, in this study we measured DNA content as nuclear area (in µm^2^) based on the positive correlation between DNA content and nuclear size [21], [57], [60], [61]. This situation has been reported previously for other algal species, where a suitable standard is yet to be found (see [62]) and it requires standard species different from those specified as appropriate for vascular plants [63].

Nuclear DNA content is referred as C-values which represent multiples of the minimum amounts of DNA corresponding to the non-replicated haploid chromosome complement [64], [65], [66]. Determination of minimum ploidy level was set in gametes of *C. prolifera*, the only Mediterranean *Caulerpa* species for which sexually reproducing fronds could be found. To assign ploidy levels to the genome size classes we followed the latest terminology f*or* genome size, C value and nuclear DNA content [67], [68].

### Statistical Analyses

**Figure 2:**
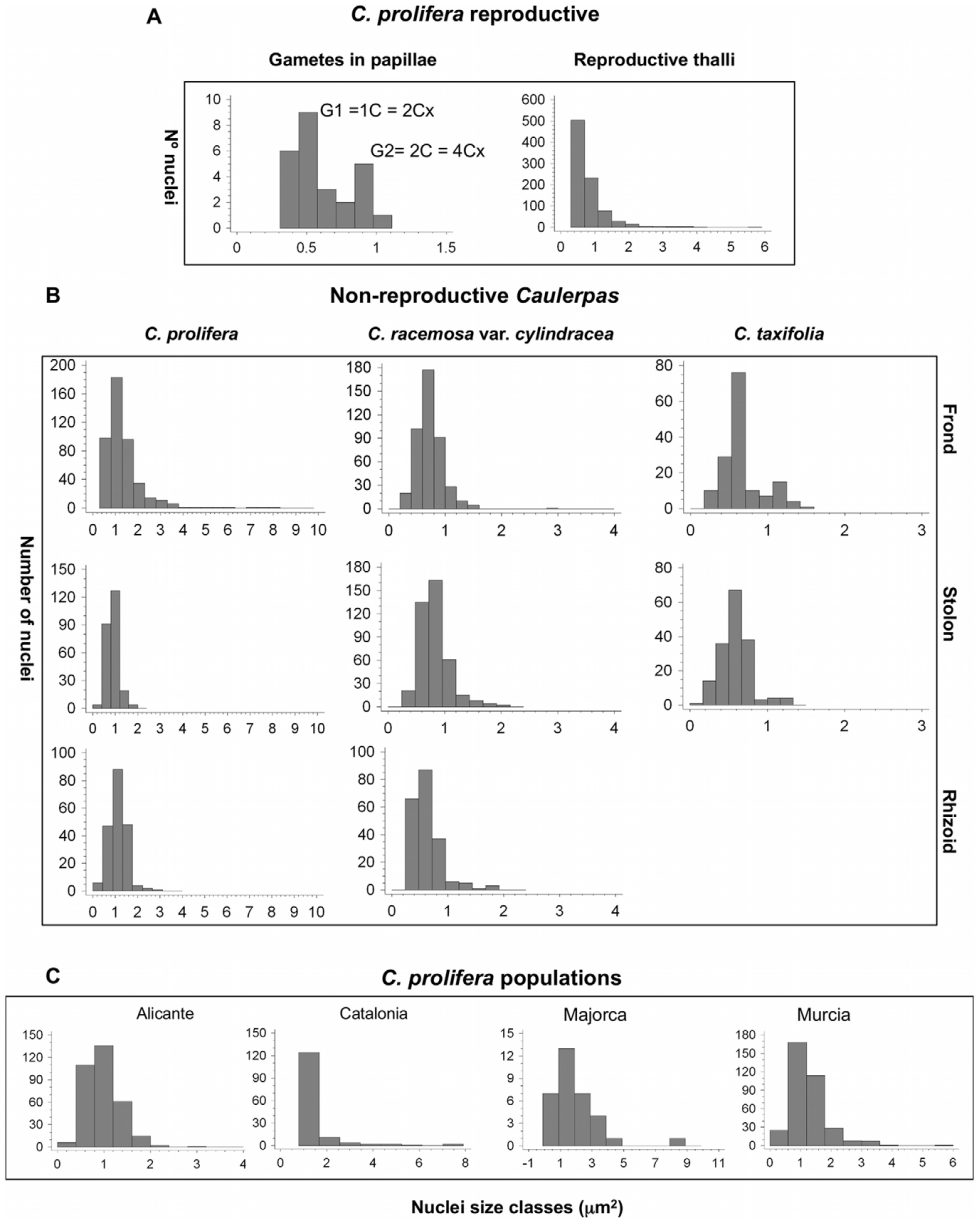
Nuclear DNA contents in *Caulerpas*. Nuclei size histograms measured from DAPI-stained DNA (correlates with genome sizes) for reproductive (A) and non-reproductive (B) species, and populations (C) of *Caulerpa* from the Mediterranean. Number of nuclei is represented in the Y-axis in all the graphs, and nuclei size classes in µm^2^ are represented in the X-axis.

For each species, population, individual or thalli portion, data was grouped into the different categories of ploidy levels according to the frequency distribution of nuclear genome sizes shown in the histograms (Fig. 2). The higher peak in each histogram was established as the G1 (unreplicated nuclei) in the corresponding ploidy level, with the following minor peak with a duplicate number as G2 (replicated nuclei). Data were then sorted into groups corresponding to the ploidy levels. For example, in gametes, G1 and G2 could be spotted clearly, with G1 set at 0.5 µm^2^ and G2 at 1.0 µm^2^, thus all the data from approximately 0.3 to 0.7 µm^2^ correspond to the first peak (G1), and from 0.71 to 1.2 µm^2^ for the second (G2). This procedure was applied for all samples. In the frond, when somatic ploidy levels overlapped, each half of the overlapping data was assigned to each of the two ploidy levels. Means and standard deviations were calculated for each group. The minimum ploidy level was set as 2Cx: G1 = 1C = 2Cx (Table 1). Statistical analyses were performed with Excel (14.2.3), Statgraphics Plus (5.1) and with GraphPad (http://graphpad.com/scientific-software/). Differences in mean genome size among groups were tested either with a *t*-test when comparing two samples or when comparing several samples with one-way analyses of variances ANOVA, all using a conservative significance level of *0.001*.

## Results

### Localization and Measurement of Nuclei in Sterile and Fertile Specimens

Localization of nuclei was a difficult task due to several factors (as described above in materials and methods). Preparations of different parts of the thalli (fronds, stolons and rhizoids) visualized by optical and fluorescence microscopy, were observed to contain bacteria, leucoplasts, chloroplasts and different epiphytes, all of which had to be distinguished from nuclei. Nuclei were smaller than plastids, and presented homogenous DAPI stained areas, whereas in the chloroplasts the DNA-containing areas were distributed round the periphery (Fig. 1). This visual identification of nuclei was in agreement with nuclear diameters (between 0.6 and 4 µm) reported in the literature for *Caulerpa* species [24], [28], [32]. Bacteria were much smaller than nuclei and presented autofluorescence, so could be easily distinguished.

Sterile and fertile fronds of *C. prolifera* were easily distinguished by marked changes in frond color, the latter having discharge tubes formed along one surface of the frond mid-axis and less frequently on the stolons (Fig. 1). These tubes were 2–3 mm long, translucent and appeared to have a yellowish or whitish plug at the apex; mucilage was released from the discharge tubes along the frond. Gametogenesis observed in this study for *C. prolifera* is similar to that previously described for other species of *Caulerpa* (holocarpic reproduction): all the cellular contents of the thallus are transformed simultaneously into numerous gametes formed inside gametangia, which are released (Fig. 1). Gametangia were two types of structures: smaller spherical ones which contained eight gametes and larger oval ones with over 60 gametes, likely corresponding respectively to female and male gametes [13], [20]. No trabeculae were observed in any liberation tube.

A total of 3156 nuclei were localized, measured and analyzed. For *C. prolifera,* 893 were from non-reproductive thalli (331 from 1 individual from Alicante, 177 from 1 individual from Catalonia, 33 from 1 individual from Majorca, 352 from 2 individuals from Murcia: 322 from 1 individual and 30 from the other), and 896 were from reproductive thalli (644 from 1 individual Catalonia, and 252 from other individual from Majorca). In non-reproductive thalli of *C. racemosa* var. *cylindracea* (Santa Pola, Alicante) 1048 nuclei could be analyzed of which 146 corresponded to 1 individual, and 902 to the other individual; and in *C. taxifolia* (France) 319, all from the same individual.

### DNA Content Levels in the Three *Caulerpa* Species

The nuclear area measurements were displayed as histograms (Fig. 2). The fluorescence emissions were in approximately a two-fold ratio, as expected, an attribution of the peaks to G1 and G2 was performed. The first peak in each histogram corresponds to the G1 DNA content, which is the highest. A minor subpopulation was evident at an intensity corresponding to the G2-phase nuclei and it could be visualized best when a higher number of nuclei are in division (in forming gametes) (Fig. 2). This pattern follows the typical eukaryotic cell cycle where most cell time is spent in G1 as also reported in other green algae [69], [70], [71].

All species exhibited variation in DNA content in all the thalli portions. According to the histograms, we calculated the mean of the minimum genome size (nuclei at G1 in the first ploidy level found) for each species/population/thalli portion (Table 1). In non-reproductive *C. prolifera*, all thalli portions presented an average minimum genome size of 1 µm^2^ independently of the location in the Mediterranean Sea: (Alicante, Catalonia, Majorca, Murcia). For *C. racemosa* var. *cylindracea* average minimum genome size was 0.7 µm^2^ and for *C. taxifolia*, 0.5 µm^2^. Genome size was very variable for *C. prolifera* and *C. racemosa* var. *cylindracea*, where nuclei reached a maximum size of 9 µm^2^ for *C. prolifera* and for *C. racemosa* var. *cylindracea,* 3 µm^2^. For reproductive thalli of *C. prolifera,* the average minimum genome size fell into a size class of 0.5 µm^2^ and nuclei reached a maximum size of 6 µm^2^. In *C. taxifolia,* average minimum genome size was 0.5 µm^2^ and no somatic ploidy was observed neither for fronds nor stolons. The data grouped in four groups for *C. prolifera*, three groups for *C. racemosa* var. *cylindracea* and two groups for *C. taxifolia*. All the nuclear sizes could be sorted in 8 groups, which were assigned to different ploidy levels (see next).

### Ploidy Levels in the Three *Caulerpa* Species

**Figure 3:**
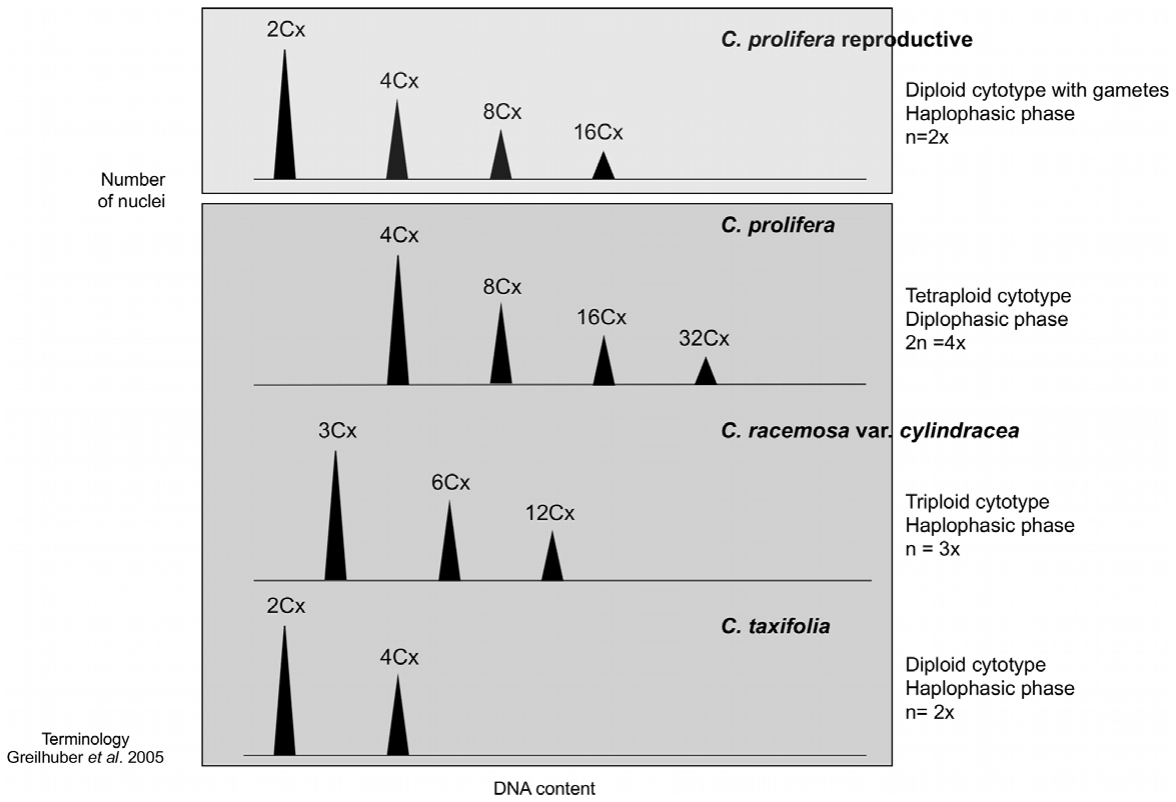
*Caulerpa* cytotypes in the Mediterranean area. Diagram of the correspondence of the peaks from DNA content histograms of cytotypes and ploidy levels using the C/Cx terminology of Greilhuber *et al*. (2005) [67], for the species found in the Mediterranean.

Assignment of ploidy levels was made first in gametes of *C. prolifera* and then in the different parts of thalli and different species, where 1C and 2C are referred as 2Cx and 4Cx ploidy levels. We assumed that gametes were unreduced (explained in discussion) and consequently DNA content would be equal to G1 = 1C = 2Cx and G2 = 2C = 4Cx (Fig. 2). Given the minimum ploidy levels in gametes, then the other peaks represent 4Cx and 8Cx nuclei, etc (Fig. 3). For *C. prolifera,* four ploidy levels were found, either sterile (4Cx, 8Cx, 16Cx and 32Cx) or reproductive (2Cx, 4Cx, 8Cx and 16Cx). Gametes found inside the reproductive papillae only have two ploidy levels (2Cx and 4Cx). Some of these ploidy levels overlapped between different nuclear sizes. *C. prolifera* is thus tetraploid, and can be either in haplophasic phase, which can produce gametes, or in diplophasic phase which does not form gametangia.

For *C. racemosa* var. *cylindracea*, three ploidy levels were found, the minimum was 3Cx (with a genome size average of 0.7 µm^2^), and the next were 6Cx and 12Cx. Thus *C. racemosa* var. *cylindracea* is a triploid because following the terminology above, the replicated nuclei value (1.5 µm^2^) at G2 was equal to three times the value of *Caulerpa* gametes (Fig. 3).

Finally for *C. taxifolia*, only two ploidy levels were found in the both thalli portions analyzed, G1 = 1C = 2Cx equal to the value found in gametes (0.5 µm^2^) and G2 = 2C = 4Cx (1.0 µm^2^). In this case, *C. taxifolia* is a diploid because the majority of the nuclei are in 2Cx. (Fig. 3).

In total, eight ploidy levels were found in *Caulerpa* species and four different cytotypes from the Mediterranean area. *C. prolifera* and *C. racemosa* var. *cylindracea* were estimated to be endopolyploids, since somatic polyploidy was found mainly in the frond for both taxa. In *C. taxifolia* no endopolyploidy was found.

### Genome Size Differences at Intra, Inter and Species Level

**Figure 4:**
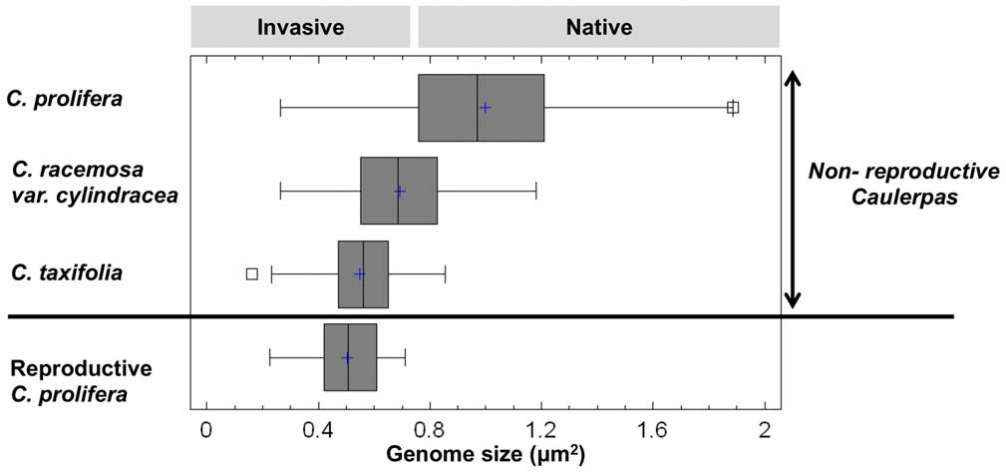
Variation in minimum genome size. Variation expressed in area (µm^2^), between non-reproductive thalli of *C. prolifera*, *C. racemosa* var. *cylindracea* and *C. taxifolia,* and reproductive *C. prolifera* (sample size n = 791, 962, 280, 517, respectively). The + near the median bar indicates location of the sample means. Genome of invasive thalli is smaller (F coefficient 603.23, *P*<*0.0001*).

Independently of ploidy level, comparison of the minimum genome size found among the three species of *Caulerpa* co-existing in the Mediterranean (Fig. 4) revealed a significant decrease (one-way ANOVA *f:* 603.23, *P<0.0001*) from *C. prolifera* to *C. racemosa* var. *cylindracea* and to *C. taxifolia* (Table 2). This significant difference was also found when comparing the full data set with all the ploidy levels included (one-way ANOVA *f:* 171.38, *P<0.0001*) (Table 3). Comparisons of the reference reproductive frond and its gametes of *C. prolifera* versus genome sizes of each species in the first ploidy level (including G1 and G2 values) revealed that sterile *C. prolifera* and *C. racemosa* var. *cylindracea* are significantly different from the reproductive *C. prolifera* (t = 23.0806, df = 1235, *P<0.0001* and t = 3.3790, df: 1241, *P<0.001* respectively), but genome sizes did not differ between nuclei from gametes of *C. prolifera* and sterile *C. taxifolia* (t = 0.1694, df: 960, *P = 0.8656*).

**Table 2:** One way ANOVA comparing the minimum genome size (G1) in the three *Caulerpa* species.

	Sum of squares	Df	Mean square	F	P value
Between groups	93.7443	3	31.2481	603.23	0.00000
Within groups	131.887	2546	0.0518015		
Total	225.631	2549			

One way ANOVAs comparing data from the thallus of sterile *C. prolifera*, sterile *C. racemosa* var. *cylindracea* and sterile *C. taxifolia,* vs. reproductive *C. prolifer*a, using the minimum genome size (G1) data.

**Table 3:** One way ANOVA comparing total data in the three *Caulerpa* species.

	Sum of squares	Df	Mean square	F	P value
Between groups	126.513	3	42.1711	171.38	0.0000
Within groups	775.6	3152	0.246066		
Total	902.113	3155			

One way ANOVAs comparing data from the thallus of sterile *C. prolifera*, sterile *C. racemosa* var. *cylindracea* and sterile *C. taxifolia,* vs. reproductive *C. prolifer*a using the full data set.

## Discussion

### Cytotypes Found in *Caulerpa* Species in the Mediterranean Sea

Our results show that the populations and species studied within the Mediterranean area are polyploids. According to the four cytotypes encountered, we conclude that in the Mediterranean Sea, both invasive *Caulerpa* species are composed of haplophasic cytotypes, triploid for *C. racemosa* var. *cylindracea* and diploid for *C. taxifolia*, whereas for the diplophasic cytotypes they would be hexaploid and tetraploid respectively (Fig. 4). The latter (diplophasic) were not observed in our Mediterranean sampling but may be present in smaller proportions in the Mediterranean; in future work we will address their role in these species in native ranges. The Mediterranean native *C. prolifera* is a tetraploid in which the dominant phase is diplophasic, and there is the second non-dominant haplophasic phase after meiosis. Evidence for a haplophasic stage of the thalli comes from the 2Cx nuclei found around the full periphery of the frond, which were considered either to represent the general nuclei of the thallus or gametes. Gametangial sacs were only found at the base of the papillae, and gametes are immediately released into the papillae as soon as released from gametangia [13], [20].

No differences among individuals of *C. prolifera* and *C. racemosa* var. *cylindracea* were found in this study, not within nor between localities. All had the same unique cytotype within a species. Genome size differences were only found at species level. Therefore we considered that sufficient individuals of each species were analyzed, besides the only one for *C. taxifolia* as this taxon in the Mediterranean arose from vegetative spread of a single invader released from the Monaco aquarium. The more than 3000 nuclei measured in this study are a much higher number than any other study of this type in algae up to date. However we cannot exclude the possibility that a much higher sample size of individuals spread across different geographical areas may reveal either the presence of different life history phases (e.g. the tetraploid or hexaploid diplophasic phase for *C. taxifolia* and *C. racemosa* var. *cylindracea*) or simply different ploidy levels for the same species.

*Caulerpa species* are polyploids in multiple ways. Besides their basal ploidy level, somatic ploidy was found in two of the species studied (*C. prolifera* and *C. racemosa* var. *cylindracea*) but not in *C. taxifolia*. This could be related to the age of the alga since specimens of *C. taxifolia* were very small at time of collection. Endopolyploidy (the multiplication of DNA and chromosomal number without nuclear division) has been reported in larger organs in crop plants (e.g. larger flowers or leaves) to ensure growth by cell enlargement in situations that prevent growth by cell division [72], [73]. Endopolyploidy was already reported in algae for Phaeophyceae [74], [75], [76], Chlorophyta [21], [77] and Rhodophyta [66], [78]. Since endopolyploidy, by multiplying the number of gene copies contributes to the mass of a growing tissue, this could be one of the strategies in *Caulerpa* for efficient clonal growth, compensating a small role of sexual reproduction in space colonization.

Our study found similar results to Kapraun [21] who found four ploidy levels within single individuals in non-reproductive *C. prolifera*, but in that study minimum ploidy level was not defined in gametes. Furthermore, we did not find an association of ploidy level to morphology within each species (cytotypes were morphologically identical within each species). Our results are in agreement with suggestions of polyploidy and hybridization for *C. racemosa* from previous studies [8], [21], [79].

### Are Gametes of *C. prolifera* Reduced or Unreduced?

The conclusions of this study are highly based on inferring whether the gametes are reduced (1Cx) or unreduced (2Cx). The available evidence indicates that gametes are not reduced since with microsatellite data (Varela-Álvarez *et al*, unpublished; [80]) we have been obtained more than two alleles at multiple loci for many samples, a result incompatible with 1Cx gametes. Also, non reduced gametes are the only suitable explanation to interpret Table 1, integrating all the eight ploidy levels detected, and where the value for replicated nuclei in *C. racemosa* var. *cylindracea* is three times the minimal genome size value set in gametes. Furthermore we can compare gametes of *C. prolifera* with nuclear sizes of *C. taxifolia* and *C. racemosa* because for the same ploidy level, nuclear sizes in *Caulerpa* have been shown to be equal across different species (as published for *Caulerpa mexicana* Sonder ex Kützing, *Caulerpa paspaloides* (Bory de Saint-Vincent) Greville, *Caulerpa verticillata* J. Agardh and *C. prolifera*); in the same ploidy level all have identical genome sizes, with 1C = 0.1 pg [21].

### Proposed Life History for *C. prolifera* in the Mediterranean Sea

**Figure 5:**
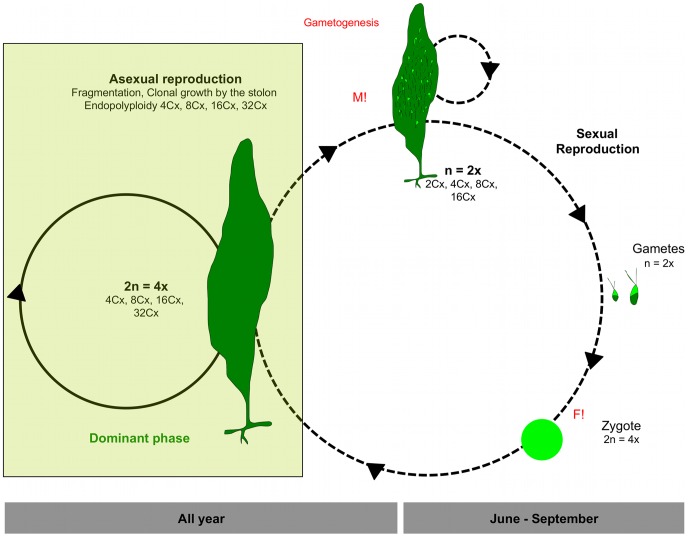
Proposed life history for *C. prolifera* in the Mediterranean Sea. (Clone in dominant phase proportionally drawn).

In this study, we propose a life history in *C*. *prolifera* as a diplophasic life cycle with only one generation involved, tetraploid (Fig. 5), in which nuclei in some thalli undergo meiosis and form gametangia. This life history is the same as observed in culture studies [25] on *Caulerpa racemosa* var. *laetevirens* Weber-van Bosse from Japan (native range) and is also in agreement with [32] who proposed that meiosis takes place during gametogenesis in the thallus. We go beyond these previous studies on the genus *Caulerpa* by determining their ploidy levels. Also we observed that during asexual propagation by clonal growth and fragmentation there is endopolyploidy occurring. The life cycle here described for *C. prolifera,* where vegetative growth takes place mainly in a diplophasic phase, which is tetraploid (with endopolyploidy), cannot explain why in the invasive taxa, *C. racemosa* var. *cylindracea* and *C. taxifolia,* only haplophasic stages were found. In these, the diplophasic phase would be hexaploid for *C. racemosa* var. *cylindracea* and tetraploid for *C. taxifolia*. This cannot be further resolved in the absence of gametes from these species, and we propose that further research should be conducted to compare Mediterranean strains of *C. racemosa* var. *cylindracea* and *C. taxifolia* with the same species in their native ranges.

Asexual reproduction via clonal fragmentation/reattachment and vegetative growth appears to be the main means of reproduction and growth of these species in the Mediterranean, or even the only means of propagation in the invasive species. While sexual reproduction of *Caulerpa* is common in tropical habitats [13], [81], [82], it is infrequent in the Mediterranean Sea. Typically, only a small number of *Caulerpa* individuals in a population become fertile during each reproductive episode, estimated as less than 20% [36] or usually 5%, but increasing occasionally to 15–20% of thalli [83]. In our study, a survey of 20 *Caulerpa* meadows along the Mediterranean separated by more than 3000 km (2008–2011; project PTDC/MAR/70921/2006, FCT, Portugal) found only a very small number of reproductive fronds of *C. prolifera,* and only in one locality (pers. observ.). In the case of *C. taxifolia* and *C. racemosa* var. *cylindracea* occasional male gametes release for the first one and female gametes release for the latter has been recorded in Croatia [83], [84]. We support the idea that Mediterranean *C. taxifolia* and *C. racemosa* var. *cylindracea* could be immature or not functional gametophytes that cloned themselves that spread via clonal propagation. In fact, it has been observed that for *Caulerpa cupressoides* (West) C. Agardh and *Caulerpa serrulata* (Forsskål) J. Agardh no macroscopic alteration of the cytoplasm (papillae formation or reorganization of cytoplasm in a net like appearance) in the erect fronds is evident when gametogenesis. However, microscopic examination of these two species at this time revealed the presence of gametes [20]. Also these authors found that some thalli presented abortive gametangia in which progressive cleavage failed to occur, and or multiple gametes arising through incomplete cleavage during gamete formation, being both types of nuclei non functional during gamete copulation. This may be the case for both invasive Mediterranean *Caulerpa* species. However, genetic evidence suggests that sex occurs in invasive *C. taxifolia* from east Australia [15], [85]. Our observation of gametes of both sexes present in the same frond indicates that *C. prolifera* is monoecious, in agreement with that found for *C. taxifolia* [13].

Variability in reproductive mode and life history traits between species and populations would have ecological and evolutionary consequences on their capacity for colonization, and on invasiveness (e.g. [86], [87], [88]). Our results show that *Caulerpa* species that are invasive in the Mediterranean spread mainly via their haplophasic phase, suggesting that this may be a favorable life history trait for invasion. One hypothesis to explain this effect could be a putatively faster replication rate for when having lower DNA content, as discussed below.

### Minimum Genome Size to Trigger Invasion in *Caulerpa* Species

Being invasive requires rapid growth rate, a trait that is correlated with low DNA amount and is not favoured by large genomes [45]. Accordingly, our results show that, regardless of ploidy levels, the minimum genome size in the invasive species (*C. taxifolia* and *C. racemosa* var. *cylindracea*) is significant smaller than in the native *C. prolifera*. The role of reductions in genome size for increasing invasiveness has been shown in a detailed analysis of DNA contents for 156 angiosperm weed species, including 97 recognized as important world weeds [89], which provided robust evidence that small genomes are a requirement for ‘‘weediness’’. Clearly, weeds appear to be characterized by possessing small genomes and once again it is apparent that having a large genome effectively limits available options. Although not all species with small genomes become invasive, weeds usually have small genomes and therefore increasing genome size might limit invasiveness potential [45].

According to our results, although *C. racemosa* var. *cylindracea* would have the largest genome size due to being hexaploid, however it was found propagating only in *a* haplophasic phase, reducing its DNA replication needs. Both *C. taxifolia* and *C. racemosa* var. *cylindracea* use the haplophasic phase (apparently as gametophytes that may produce gametes or not, including reported unviable gametes) to proliferate in the Mediterranean, becoming invasive (Fig. 4).

### Evolution of Ploidy Levels and Genome Sizes vs. Invasion Strategies

Invasive behavior appears to be positively correlated with ploidy level [39, 90]. Why polyploids are overrepresented on lists of invasive species is currently unknown, although their generally higher heterozygosity might increase ecological success in many ways [91], [92]. This might be particularly important to counteract the loss of diversity created by low sexual recombination in highly clonally propagating populations. The advantages of polyploidy [93], [94], [95] are more obvious for allopolyploids, in which alleles of two or more species are combined [96], increasing genetic diversity among such polyploid complexes. A hybrid origin of a Mediterranean *C. racemosa* strain [16] suggests the hypothesis of an allopolyploid origin, which may contribute to its invasive success. In the last 17 years, *C. racemosa* colonized 12 countries and all major islands in the Mediterranean as well as the Canary Islands in the Atlantic [9], [11], an invasive potential that surpasses the weedy strain of *C. taxifolia* [97]. In plants, newly formed polyploids and particularly those of hybrid origin (allopolyploids) are frequently invasive [90]. Allopolyploidy may confer immediate ecological aptitude to invade new habitats thereby fostering invasiveness [98], [99]. This is the case of several allopolyploid plants throughout the world [100], [101], [102], [103], [104].

It is known that four types of evolutionary change that might promote rapid evolution in the introduced range: bottlenecks, hybridization, polyploidy, and stress-induced modification of the genome [105]. It would be of great interest to determine if evolution in this group has been accompanied by transformations involving chromosome complements and nuclear DNA contents. Future research should look for sources of polyploidy during the evolutionary history of this genus as a contribution towards understanding what creates new invaders.

### Conclusions

Our cytogenetic data elucidated ploidy levels in three *Caulerpa* species and allowed us to propose hypotheses for their life histories and invasion strategies in the Mediterranean Sea. We propose for *C. prolifera* in the Mediterranean a diplophasic life cycle with only one generation involved which is tetraploid. For *C. racemosa* var. *cylindracea* and *C. taxifolia*, clones in haplophasic phase dominate in the Mediterranean. *C. racemosa* var. *cylindracea* is triploid and *C. taxifolia* is diploid in this area. Somatic ploidy was characteristic of *C. prolifera* and *C. racemosa* var. *cylindracea* but not of *C. taxifolia*. We suggest that vegetative propagation by means of the phase with reduced genome size (haplophasic) and the polyploidy, possibly allopolyploidy in *C. racemosa* var. *cylindracea*, all contribute to their success as invasive strains. We also postulate that life histories of *Caulerpa* species may be flexible, and these may present different ploidy levels and different phase dominance in other regions outside the Mediterranean Sea.
